# Serotonergic Contributions to Human Brain Aggression Networks

**DOI:** 10.3389/fnins.2019.00042

**Published:** 2019-02-22

**Authors:** Martin Klasen, Dhana Wolf, Patrick D. Eisner, Thomas Eggermann, Klaus Zerres, Florian D. Zepf, René Weber, Klaus Mathiak

**Affiliations:** ^1^Department of Psychiatry, Psychotherapy and Psychosomatics, Faculty of Medicine, RWTH Aachen, Aachen, Germany; ^2^JARA – Translational Brain Medicine, Aachen, Germany; ^3^Institute of Human Genetics, Medical School, RWTH Aachen University, Aachen, Germany; ^4^Department of Child and Adolescent Psychiatry, Psychosomatic Medicine and Psychotherapy, Jena University Hospital, Friedrich Schiller University, Jena, Germany; ^5^Centre and Discipline of Child and Adolescent Psychiatry, Psychosomatics and Psychotherapy, Division of Psychiatry and Clinical Neurosciences and Division of Paediatrics and Child Health, School of Medicine, Faculty of Health and Medical Sciences, University of Western Australia, Perth, WA, Australia; ^6^Telethon Kids Institute, Perth, WA, Australia; ^7^Media Neuroscience Lab, Department of Communication, University of California, Santa Barbara, Santa Barbara, CA, United States

**Keywords:** serotonin, aggression, amygdala, tryptophan depletion, PFC, supramarginal gyrus

## Abstract

Aggressive behavior is associated with dysfunctional frontolimbic emotion regulation circuits. Recent findings suggest serotonin as a primary transmitter for prefrontal amygdala control. However, the association between serotonin levels, amygdala regulation, and aggression is still a matter of debate. Neurobehavioral models furthermore suggest a possible mediating influence of the monoamine oxidase A gene (*MAOA*) on this brain-behavior relationship, with carriers of low expressing allele varieties being a risk group for aggression. In the present study, we investigated the influence of brain serotonin modulation and *MAOA* genotype on functional amygdala connectivity during aggressive behavior. Modulation of serotonergic neurotransmission with acute tryptophan depletion (ATD) and placebo were administered in a double-blind, cross-over design in 38 healthy male participants. Aggressive behavior was modeled in a violent video game during simultaneous assessment of brain activation with functional magnetic resonance imaging (fMRI). Trait aggression was measured with the Buss-Perry Aggression Questionnaire (BP-AQ), and *MAOA* genotypes were assessed from blood samples. Voxel-wise functional connectivity with anatomically defined amygdala was calculated from the functional data. Tryptophan depletion with ATD reduced aggression-specific amygdala connectivity with bilateral supramarginal gyrus. Moreover, ATD impact was associated with trait aggression and *MAOA* genotype in prefrontal cortex regions. In summary, serotonergic corticolimbic projections contribute to aggressive behavior. Genotype-specific vulnerability of frontolimbic projections may underlie the elevated risk in low expressing allele carriers.

## Introduction

Aggression is associated with dysregulation in a corticolimbic network (Davidson et al., 2000; Siever, 2008). Specifically, a deficient regulation of the amygdala via prefrontal cortex (PFC) areas has been described as a risk factor for aggressive behavior (Coccaro et al., 2011). The PFC-amygdala system supports affective control (Lederbogen et al., 2011) and regulates aggressive impulses (Bufkin and Luttrell, 2005). Evidence for this corticolimbic aggression model comes from both structural and functional neuroimaging studies. Furthermore, reduced gray matter in PFC and inferior temporal lobe including the amygdala has been related to antisocial traits (Ermer et al., 2012; Gregory et al., 2012). In a similar vein, reduced PFC-amygdala connectivity has been described as a risk factor for trait aggression in patients with schizophrenia (Hoptman et al., 2010). In healthy individuals, enhanced amygdala activation is a neural substrate of state anger after provocation (Yu et al., 2014), whereas lateral PFC activation counteracts aggressive reactions in such situations (Achterberg et al., 2016). In summary, functioning of the PFC-amygdala regulation system seems to be central to successful emotion regulation (Banks et al., 2007), thus preventing impulsive aggression.

On the transmitter level, aggression has frequently been associated with alterations of serotonergic neural activity (Duke et al., 2013). Pharmacological serotonin challenges have been reported to influence both PFC-amygdala connectivity and aggressive feelings (Klasen et al., 2013). One established method to challenge serotonergic activity specifically is acute tryptophan depletion (ATD). In short, ATD is a neurodietary method which temporarily reduces serotonin levels via a modification of the precursor tryptophan (see Hood et al., 2005, for an overview). Lowered serotonin levels after ATD lead to increased aggression (Bjork et al., 1999; Kötting et al., 2013). On the neural level, ATD modulates functional connectivity between amygdala and lateral PFC areas (Eisner et al., 2017). Specifically, ATD challenge has been reported to reduce the processing of aggression-relevant stimuli in the amygdala-PFC system (Passamonti et al., 2012). Remarkably, the impact of ATD on aggression-related amygdala connectivity seems to depend on personality traits, with higher reward drive being associated with a larger ATD impact (Passamonti et al., 2012). The relationship, however, between ATD impact on amygdala connectivity and aggressive traits remains unknown so far.

Besides dietary and pharmacological challenges, genetic factors influencing brain serotonin levels have been associated with aggression as well. A prominent example is the Brunner syndrome, a rare genetic mutation which causes a functional knockout of the monoamine oxidase A (MAO-A) gene (*MAOA*) and is associated with higher levels of aggression and delinquency (Brunner et al., 1993). The enzyme MAO-A degrades serotonin (Shih et al., 1999), with a complete knockout leading to an excess in brain serotonin levels. Non-pathological variations in serotonin transmission caused by an upstream variable number tandem repeat (uVNTR) polymorphism of the *MAOA* gene have been associated with aggression as well (Pavlov et al., 2012). Specifically, a gene-environment interaction has been proposed as a risk factor in male carriers of low expressing gene variants (*MAOA*-L). *MAOA*-L carriers have a high vulnerability to develop aggressive behavior after the experience of childhood trauma than carriers of high expressing gene variants (*MAOA*-H, Caspi et al., 2002). An established neurobiological model proposes a reduced amygdala regulation due to a blunted serotonergic system as a neurobiological endophenotype in these risk allele carriers (Buckholtz et al., 2008). Recent findings show that trait aggression networks vary as a function of *MAOA* genotype (Klasen et al., 2018), but the role of serotonin in this brain-behavior relationship is still a matter of debate.

The present study investigated serotonergic influences on amygdala connectivity associated with aggressive behavior in 38 healthy males. Aggressive behavior was modeled via virtual reality in a violent video game. Violent video game tasks activate similar neurobiological networks compared to behavioral aggression tasks and real-life aggression (Mathiak and Weber, 2006; Weber et al., 2006). Transfer effects between playing realistic and salient violent video games and real-life aggression have been demonstrated in both experimental and longitudinal behavioral studies (Prescott et al., 2018). Thus, a violent video game task can serve as a valid aggression model and has been employed in various neuroimaging studies on aggression (e.g., Klasen et al., 2013; Zvyagintsev et al., 2016; Wolf et al., 2018). Serotonin levels were manipulated via ATD challenge in a double-bind, randomized, and placebo-controlled design. Moreover, genetic differences in serotonin effects were assessed by *MAOA* genotyping. The *MAOA* gene is located on the X chromosome; accordingly, a number of studies confirmed that genotype influences on behavior as well as on neural networks is stronger in men than in women (Kim-Cohen et al., 2006; Buckholtz et al., 2008; Buckholtz and Meyer-Lindenberg, 2008; Guo et al., 2008; Edwards et al., 2010). Following these findings, only male participants were included in the present study.

Based on previous findings (Eisner et al., 2017), we expected a reduction of aggression-specific amygdala-PFC connectivity by ATD. Moreover, we hypothesized this reduction to be associated with trait aggression, i.e., a larger ATD impact in more aggressive individuals. Finally, we sought to explore genotype influences on ATD impact. Following the assumption of enhanced neurobiological vulnerability, we expected a larger ATD effect on amygdala-PFC coupling in male carriers of low expressing *MAOA* variants (*MAOA*-L; risk allele carriers).

## Materials and Methods

### Participants

Thirty eight male Caucasians (16–33 years, mean age 24.7 ± 3.6 years) participated in the study. All participants had normal or corrected to normal vision, normal hearing, no contraindications against MR investigation, no history of neurological or psychiatric illness according to the SCID screening questionnaire (Wittchen et al., 1997), and no history of psychopharmacological therapy. All participants were right-handed according to the Edinburgh Handedness Inventory (Oldfield, 1971). The experiment was designed according to the Code of Ethics of the Word Medical Association (Declaration of Helsinki, 2013), and the study protocol was approved by the local Ethics Committee. After detailed briefing and instruction, participants gave written informed consent.

### Serotonin Challenge

The present study employed a randomized double-blind and placebo-controlled cross-over design. Each participant was measured in an ATD and a placebo condition, taking place on separate days. Findings from a third condition (escitalopram) have previously been reported in Wolf et al. (2018). The measurements were separated by at least 1 week allowing for a sufficient washout. Condition order was randomized across participants. The ATD condition consisted of a body weight-adapted ATD drink according to the Moja-De scheme (Moja et al., 1988; Demisch et al., 2002). Placebo consisted of a tryptophan-balanced drink (PLAC) with no tryptophan depletion effect. For ATD, a tryptophan-free amino acid beverage was applied: for 10 kg body weight 0.084 g L-Isoleucine (ILE), 0.132 g L-Leucine (LEU), 0.12 g L-Lysine-HCL (LYS), 0.05 g L-Methionine (MET), 0.132 g L-Phenylalanine (PALA), 0.06 g L-Threonine (THR), and 0.096 g L-Valine (VAL). The PLAC mixture included additional 0.7 g L-Tryptophan (TRP) per 10 kg body weight and thus had no impact on 5-HT synthesis in the brain (Biskup et al., 2012). Administration order was block-randomized (block size 6). Amino acids were packed in coded containers by a person not further involved in the experimental procedure. Administration took place at the beginning of each measurement day (about 08:30 am). Functional measurements were conducted after a delay of 3 h, allowing the serotonin challenge to take effect.

### Genotyping

Prior to the fMRI session, all participants underwent a 9 ml venous blood sampling. Genomic DNA was isolated from peripheral lymphocytes with a routine salting-out procedure. For the determination of the *MAOA* genotype, standard polymerase chain reaction (PCR) amplification was performed in a 25-μl volume containing 80 ng of genomic DNA, 1 unit of recombinant Taq polymerase (Invitrogen, Darmstadt/Germany), PCR buffer (10 mM Tris-HCl, 50 mM KCl, 2.5 mM MgCl 2, pH 8.3), 200 mM dNTPs, and 20 pmol of each primer. *MAOA* primers were obtained from Sabol et al. (1998). The PCR was run on an MJ PTC200 Temperature Cycler (Biozym, Hessisch-Oldendorf, Germany), and each of the 35 cycles consisted of a 95°C denaturation step for 45 s, a 62°C annealing step for 30 s, and, finally, a 72°C elongation step for 90 s. PCR products were run on an automated sequencing system (AB3130, Applied Biosystems, Darmstadt, Germany), and the electropherograms were analyzed with gene mapping software. According to the established classification of Sabol et al. (1998), 3.5 and 4 repeats were classified as representing a high MAO-A expression (*MAOA*-H) and 3 and 5 repeats as representing a low expression (*MAOA*-L).

### Data Acquisition

During four scanning session (310 volumes each), the participants played two versions of the racing game Carmageddon: TDR 2000 (Torus Games, Bayswater, Australia, 2000) in an unrestricted manner. In its violent version, the participants were instructed to kill as many pedestrians as possible by hitting them with their car. Hitting pedestrians induced excessive blood splatter and screaming of the victims. In the non-violent version, the players’ task was to hit colorful icons with their car; the absence of pedestrians in this game version did not allow for violent interactions. Hitting icons was accompanied by color explosion and by sound. Visual and auditory stimulation levels of the violent and non-violent game versions were comparable. Participants played the game with their right hand, using an MR-compatible 5-button keyboard. Visual stimulation and game sound were delivered via MR-compatible video goggles and headphones; sound levels were adjusted individually to a comfortable level. Participants played two violent and two non-violent sessions in randomized order on each measurement day. In combination with the serotonergic modulation, four conditions emerged, all of them session-wise with two sessions each [ATD Violence, ATD Non-Violence, PLAC(ebo) Violence, and PLAC Non-Violence].

Functional MRI was conducted on a 3 Tesla MR Scanner (Magnetom Trio, Siemens) with a 12-channel head coil using echo-planar imaging (EPI) sequences (TE = 28 ms, TR = 2000 ms, flip angle = 77°, voxel size = 3 × 3 mm, matrix size = 64 × 64, 34 transverse slices, 3 mm slice thickness, 0.75 mm gap). On each measurement day, 1240 functional images were obtained (4 sessions with 310 volumes). After completing the functional measurements, high-resolution T1-weighted anatomical images were performed using a magnetization prepared rapid acquisition gradient echo (MPRAGE) sequence (TE = 2.52 ms; inversion time TI = 900 ms; TR = 1900 ms; flip angle = 9°; FOV = 256 × 256 mm^2^; 1 mm isotropic voxels; 176 sagittal slices). Total scanning time (including preparations) was about 1 h.

On the first measurement day, all participants completed a validated German Version of the Buss-Perry Aggression Questionnaire (BP-AQ; Herzberg, 2003). The BP-AQ is a well-established 29-item self-report inventory for trait aggression, based on an empirically validated 4-dimensional model of aggression (Physical Aggression, Verbal Aggression, Anger, and Hostility). The BP-AQ was assessed directly after ATD or PLAC intake; thus, there was a time lag of ∼3 h between questionnaire and functional measurement. Only total BP-AQ scores were considered for the analyses.

### Data Analysis

Questionnaires and demographical data were analyzed with SPSS 25 (IBM Corp., Armonk, NY, United States). Functional and anatomical image analysis was performed with the SPM toolbox *CONN* (version 17.b; Whitfield-Gabrieli and Nieto-Castanon, 2012). Preprocessing included slice time correction, 3D motion correction, Gaussian spatial smoothing (4 mm FWHM), and high-pass filtering including linear trend removal. The first five images of each session were discarded to avoid T1 saturation effects. Functional images were co-registered to anatomical data and normalized by transformation into MNI space. According to the standard procedures for the removal of confounders in the CONN toolbox, 12 motion parameters (rigid body transformations and their first-order derivatives) and individual time courses from white matter and cerebrospinal fluid were extracted and regressed out of the image time series. Data analysis was restricted to a whole-brain mask in MNI space.

In a second level analysis we assessed condition-specific connectivity patterns. Four conditions entered the second level analysis: ATD Violence, ATD Non-Violence, PLAC Violence, and PLAC Non-Violence. Condition-specific connectivity was assessed over the time course of the entire session. As seed region, we employed bilateral amygdala as defined by the SPM Anatomy Toolbox (Eickhoff et al., 2005).

To assess aggression-specific amygdala connectivity in the placebo condition, we first mapped the contrast (PLAC Violence > PLAC Non-Violence). The ATD modulation of aggression-specific amygdala connectivity was then determined by the contrast (ATD Violence > ATD Non-Violence) > (PLAC Violence > PLAC Non-Violence).

Neural networks of state aggression may depend on aggressive traits as well. We therefore assessed if connectivity patterns in the above mentioned contrasts varied as a function of trait aggression. For this analysis, we correlated voxel-wise contrast values with individual BP-AQ scores, as implemented in the CONN toolbox. Finally, two samples *t*-tests were calculated to explore possible influences of *MAOA* genotypes on the above mentioned contrasts. We expected drug effects on amygdala connectivity being rather distributed and therefore allowed for a locally rather lenient threshold but a strict global correction for false discoveries. Therefore the precise locations of activation in emerging clusters need to be interpreted with caution (cf. Woo et al., 2014). All contrasts were thresholded at a voxel-wise *p* < 0.01 and corrected for multiple comparisons using false discovery rate (FDR) correction on the cluster level (*p* < 0.05).

## Results

### ATD Modulation of Aggression-Specific Amygdala Connectivity

**FIGURE 1:**
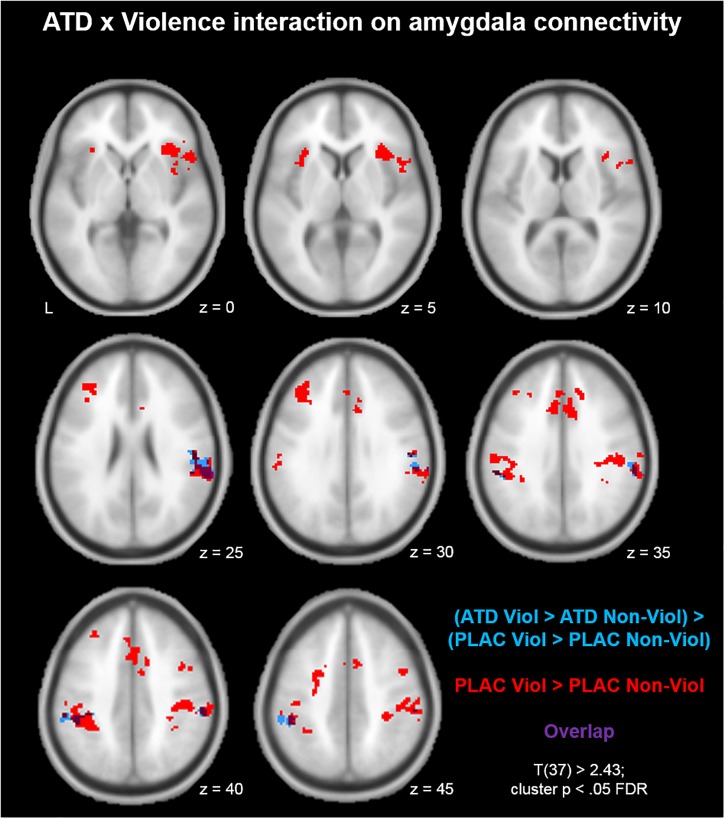
Acute tryptophan depletion (ATD) modulation of aggression-specific amygdala connectivity. Compared to the non-violent modification, the violent game increased amygdala connectivity with bilateral supramarginal gyrus (SMG), bilateral anterior insula, dorsal ACC, left middle frontal gyrus (MFG), and somatosensory cortex (red clusters). ATD attenuated this effect in bilateral SMG (blue clusters; overlap of the contrasts shown in purple).

First, we mapped brain areas that showed aggression-specific amygdala connectivity in the placebo condition with the contrast (PLAC Violence > PLAC Non-Violence; Figure 1, red clusters). Clusters from this contrast are described in Table 1 Red, including peak voxel MNI coordinates, peak t values, and cluster size. Aggression-specific amygdala connectivity encompassed bilateral supramarginal gyrus (SMG), bilateral anterior insula, dorsal ACC, left middle frontal gyrus (MFG), and somatosensory cortex (Figure 1, red). ATD modulation of aggression-specific amygdala connectivity was observed in bilateral SMG, as determined by the comparison (ATD Violence > ATD Non-Violence) > (PLAC Violence > PLAC Non-Violence). This contrast produced negative clusters only, i.e., ATD reduced aggression-specific amygdala connectivity in these regions (Figure 1, blue clusters and Table 1, Blue). Increases in aggression-specific amygdala connectivity after ATD were not observed. ATD modulation effects were restricted to aggression-specific areas found in the violence contrast and adjacent areas of the same anatomical structures (Figure 1, overlap shown in purple).

**Table 1:** Clusters from Figure 1.

Cluster	Brain region	MNI coordinates				
		*x*	*y*	*z*	Peak *T*	*k_E_*
**Red**						
1	Supramarginal gyrus r, superior parietal lobule r, postcentral gyrus r	34	-44	62	5.28	1207
2	Supramarginal gyrus l, superior parietal lobule l, postcentral gyrus l	-56	-24	34	4.84	776
3	Inferior frontal gyrus r, anterior insula r	34	26	4	5.53	491
4	SMA r/l	12	2	72	5.89	405
5	Dorsal ACC r/l, paracingulate gyrus r/l	0	16	40	4.44	402
6	Superior frontal gyrus l, Precentral gyrus l	-24	-16	60	4.27	338
7	Middle frontal gyrus l	-38	32	32	4.94	255
8	Anterior insula l	-30	22	4	4.29	141
9	Cerebellum l	-34	-52	-34	4.67	109
10	Middle frontal gyrus l	46	2	48	4.57	99
**Blue**						
1	Supramarginal gyrus r	58	-32	28	-4.80	373
2	Supramarginal gyrus l	-56	-34	40	-4.84	162


Considering potential priming effects following a violence exposure, it seems plausible that the pattern of participants starting with a violent session differed from the pattern of those participants starting with a non-violent session. To rule out such order effects, we compared the two groups on the above mentioned contrasts in additional independent samples t tests. No group differences were observed for these comparisons, neither for aggression-specific amygdala connectivity nor for its ATD modulation.

### ATD Modulation of Aggression-Specific Amygdala Connectivity: Correlation With Trait Aggression

**FIGURE 2:**
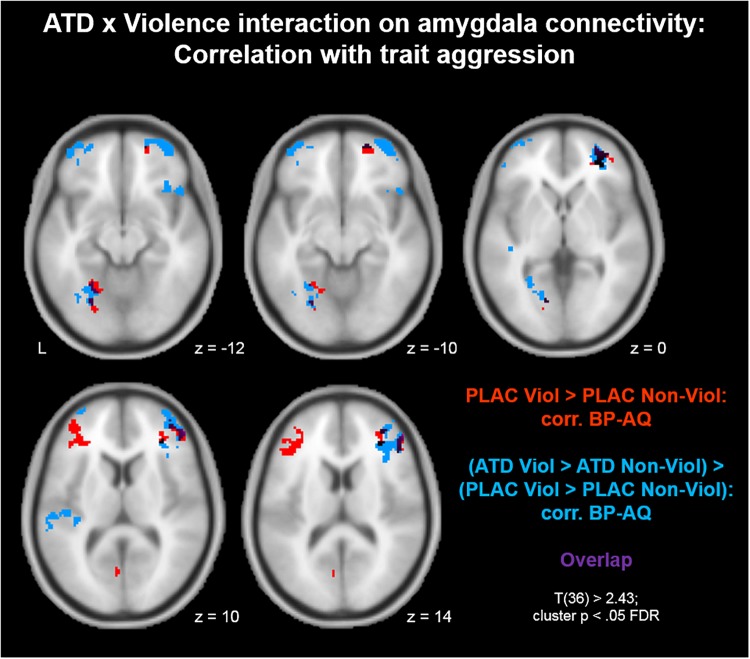
Acute tryptophan depletion modulation of aggression-specific amygdala connectivity: Correlation with trait aggression. Positive correlations between amygdala connectivity and trait aggression were observed in bilateral inferior (IFG) and middle frontal gyri (MFG; red clusters). Similarly, ATD modulation of aggression-specific amygdala connectivity correlated with trait aggression as well. Negative correlations between contrast values and BP-AQ scores were observed in bilateral orbitofrontal cortex (OFC), IFG and MFG, left fusiform gyrus (FFG), parieto-occipital areas, and left auditory cortex (blue clusters). ATD attenuation effects on amygdala connectivity with these areas were larger in more aggressive individuals. Both contrasts overlapped in right OFC, right IFG, and left FFG (purple).

To investigate whether aggression-specific amygdala connectivity patterns varied as a function of trait aggression, we correlated voxel-wise interaction values of the contrast (PLAC Violence > PLAC Non-Violence) with individual BP-AQ scores. Positive correlations between amygdala connectivity and trait aggression were observed in bilateral inferior (IFG) and middle frontal gyri (MFG; Figure 2, red clusters). Clusters from this contrast are described in Table 2 Red. Moreover, we investigated whether the ATD modulation of aggression-specific amygdala connectivity [(ATD Violence > ATD Non-Violence) > (PLAC Violence > PLAC Non-Violence)] correlated with trait aggression as well. Negative correlations between contrast values and BP-AQ scores were observed in bilateral orbitofrontal cortex (OFC), IFG and MFG, left fusiform gyrus (FFG), parieto-occipital areas, and left auditory cortex (Figure 2, blue and Table 2, Blue). Positive correlations were not observed. A substantial overlap of both contrasts was observed in right OFC, right IFG, and left FFG (Figure 2, purple). Amygdala connectivity with these areas was thus stronger reduced by ATD in more aggressive individuals.

**Table 2:** Clusters from Figure 2.

Cluster	Brain region	MNI coordinates				
		*x*	*y*	*z*	Peak *T*	*k_E_*
**Red**						
1	Inferior frontal gyrus l, middle frontal gyrus, orbitofrontal cortex l	-54	34	18	4.93	375
2	Inferior frontal gyrus r, middle frontal gyrus r, orbitofrontal cortex r	20	56	-10	4.76	334
3	Superior parietal lobule l	-24	-62	52	5.40	183
4	Fusiform gyrus l, lingual gyrus l	-20	-76	-6	4.02	161
5	Cerebellum r	8	-44	-36	5.35	142
6	Lingual gyrus l, cerebellum l	-24	-56	-12	4.34	120
**Blue**						
1	Inferior frontal gyrus r, middle frontal gyrus r	48	34	18	-4.81	745
2	Fusiform gyrus l	-34	-76	-20	-4.98	498
3	Orbitofrontal cortex l	-42	52	-16	-4.38	267
4	Orbitofrontal cortex r	34	60	-12	-5.24	206
5	Cerebellum r	4	-82	-36	-4.38	169
6	Superior parietal lobule l	-28	-58	50	-3.92	165
7	Heschl’s gyrus l, superior temporal gyrus l	-60	-30	8	-4.01	153
8	Lateral occipital cortex l	-6	-82	48	-3.50	131
9	Inferior frontal gyrus r, middle frontal gyrus r	50	18	30	-3.99	126
10	Anterior insula r	48	22	-12	-4.80	113


Aggression in general and BP-AQ scores in specific have been shown to decline with age (e.g., Redondo et al., 2017). To rule out any potential confounding effects of participant age on aggression-specific amygdala connectivity and the ATD modulation of the latter, we performed an additional mapping of the contrasts described in Figure 2 and controlled for age as a covariate. Results from Figure 2 could be replicated in almost identical fashion. Thus, neither aggression networks nor their serotonergic modulation were biased by age effects.

**FIGURE 3:**
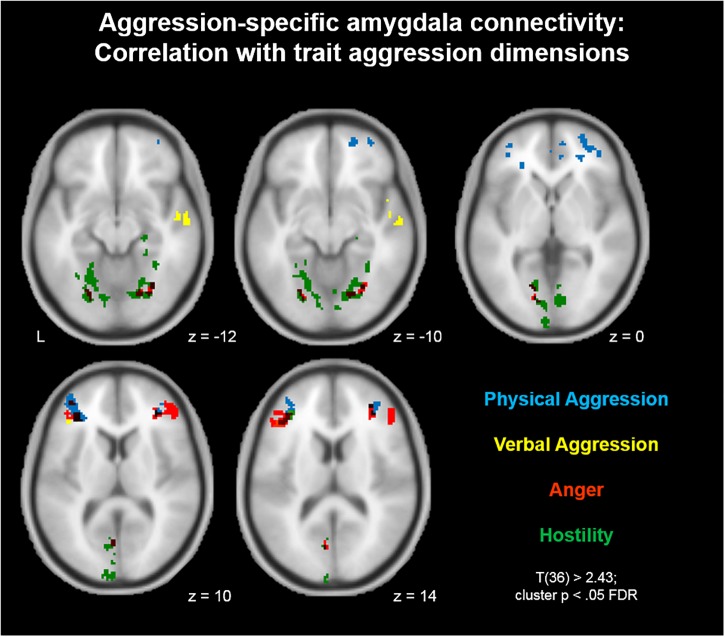
Aggression-specific amygdala connectivity: Correlation with trait aggression dimensions. The correlation between aggression-specific amygdala connectivity and trait aggression was mapped separately for all BP-AQ dimensions. Similar correlations in IFG and MFG were observed for all four dimensions, although most prominently for Physical Aggression (blue) and Anger (red). More dimension-specific correlations were observed for visual (Anger/Hostility) and auditory (Verbal Aggression) processing streams.

For a better understanding of the relationship between aggression-specific amygdala connectivity and trait aggression, we furthermore mapped this contrast separately for all four dimensions of the BP-AQ (Physical Aggression, Verbal Aggression, Anger, and Hostility). The results are depicted in Figure 3. In summary, correlations in IFG and MFG were similar for all four dimensions, although the most prominent contributions could be assigned to the dimensions Physical Aggression (blue) and Anger (red). More dimension-specific correlations were observed for visual (Anger/Hostility) and auditory (Verbal Aggression) processing streams.

### ATD Modulation of Aggression-Specific Amygdala Connectivity: *MAOA* Effects

Finally, we explored putative *MAOA* genotype differences regarding the ATD impact on aggression-specific amygdala connectivity. After genotyping, 11 participants (29%) were classified as *MAOA*-L carriers and 27 participants (71%) were classified as *MAOA*-H carriers, which is in line with previously reported gene frequency distributions in male Caucasian populations (Sabol et al., 1998). Allele frequencies are reported in Table 3.

**Table 3:** *MAOA* uVNTR allele frequencies.

	Number of repeats			
	3	3.5	4	5
Number of participants	10	2	25	1


**FIGURE 4:**
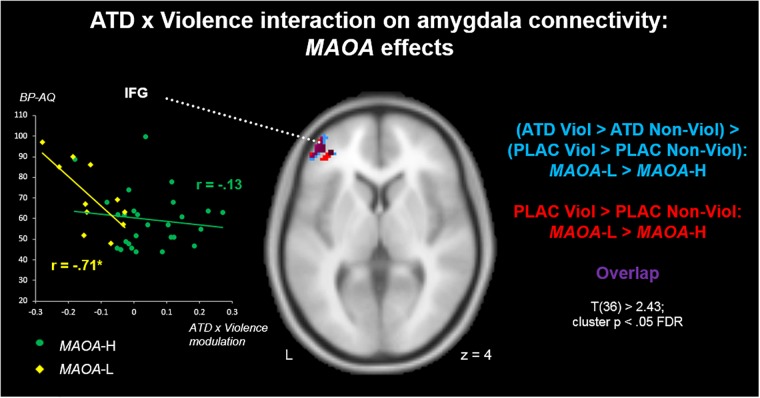
ATD modulation of aggression-specific amygdala connectivity: *MAOA* effects. *MAOA*-L carriers had stronger aggression-specific amygdala connectivity patterns with left IFG than *MAOA*-H carriers (red). Also, this pattern was more susceptible to ATD-induced connectivity reductions in *MAOA*-L than in *MAOA*-H carriers (blue). Correlations of ATD effects with BP-AQ scores highlighted a functional significance of serotonergic IFG-amygdala projections for trait aggression in *MAOA*-L, but not *MAOA*-H carriers (left insert).

A comparison of the two genotypes (*MAOA*-L > *MAOA*-H) on the contrast (PLAC Violence > PLAC Non-Violence) revealed a stronger amygdala connectivity with left IFG for the *MAOA*-L carriers (Figure 4, red clusters). Clusters from this contrast are described in Table 4 Red. A genotype comparison on the ATD impact (ATD Violence > ATD Non-Violence) > (PLAC Violence > PLAC Non-Violence) showed a stronger attenuation effect (reduced connectivity) for the *MAOA*-L group in the left IFG (Figure 4, blue clusters and Table 4, Blue). Both contrasts yielded highly similar clusters with a substantial overlap in left IFG (Figure 4, purple clusters). The reversed comparison (*MAOA*-H > *MAOA*-L) yielded no significant results for both contrasts.

**Table 4:** Clusters from Figure 4.

Cluster	Brain region	MNI coordinates				
		*x*	*y*	*z*	Peak *T*	*k_E_*
**Red**						
1	Inferior frontal gyrus l	-42	50	06	4.88	191
**Blue**						
1	Inferior frontal gyrus l	-34	46	8	-4.37	185


To explore a possible relevance of genotype differences in serotonergic modulation for aggression, we moreover correlated values from the ATD cluster (blue) with BP-AQ scores separately for both genotype groups. On average, *MAOA*-L carriers had higher BP-AQ scores than *MAOA*-H carriers [mean scores 70.64 vs. 59.52; t(36) = 2.13, *p* < 0.05]. We found a significant correlation between ATD modulation and trait aggression in the *MAOA*-L carriers [*r*(9) = -0.71; *p* < 0.05], with a stronger connectivity reduction via ATD (negative values) being associated with higher aggression (Figure 4, yellow). This effect was absent in the *MAOA*-H carriers [*r*(25) = -0.13; *p* = 0.52; Figure 4, green]. However, genotype differences in correlation coefficients (cf. Weaver and Wuensch, 2013) reached only trend level (*p* = 0.07) and must therefore be considered descriptive.

## Discussion

The present study investigated serotonergic modulation effects of aggression-specific amygdala connectivity using ATD. We found an ATD impact on aggression-specific amygdala connectivity in bilateral SMG. Moreover, serotonergic modulation ability varied as a function of trait aggression in PFC regions, with higher aggression predicting a stronger ATD impact during virtual violence. Interaction effects in PFC were stronger in *MAOA*-L compared to *MAOA*-H carriers, emphasizing their neurobiological vulnerability as a risk group for aggressive behavior.

The topography of ATD induced clusters was largely limited to SMG areas with a aggression-specific connectivity pattern. Previous research shows that SMG connectivity is susceptible to serotonergic challenge with ATD (Helmbold et al., 2016). Functionally, the SMG supports Theory of Mind abilities (Saxe and Powell, 2006) and is a core region in the brain’s empathy network (Shamay-Tsoory, 2011). As such, it is an important hub in the perception of socio-affective stimuli (Göttlich et al., 2017). Functional impairments of the SMG reduce an individual’s pain empathy toward others (Coll et al., 2017). Accordingly, the SMG plays a role in aggression and violence as well. Decreased activity in a fronto-parietal network encompassing the SMG has been associated with desensitization toward violent media (Strenziok et al., 2011). Similar findings have been obtained for the amygdala. During virtual violence, amygdala activity is attenuated, indicating a suppression of the emotional response in favor of the cognitive aspects of the task (Mathiak and Weber, 2006; Weber et al., 2006). Reduced coupling of the SMG with the amygdala after ATD is also in line with findings from neuroimaging genetics, showing that the role of the SMG in aggression-related networks depends on genes influencing serotonin transmission. Specifically, a coupling of the SMG with limbic areas seems to counteract aggressive impulses (Klasen et al., 2018). Reduced connectivity between SMG and amygdala may thus be a neurobiological substrate of increased aggression after ATD (Bjork et al., 1999; Kötting et al., 2013).

Aggression-specific amygdala networks were convergent with findings from other aggression studies and corroborate the validity of the present paradigm. Aggression-specific amygdala networks involved MFG and the nodes of the salience network in anterior insula and dorsal ACC (Seeley et al., 2007). The involvement of the salience network in aggression is well established; in specific, enhanced activity in a network of amygdala, anterior insula, and dorsal ACC is a characteristic signature of state aggression (Yu et al., 2014). Blair (2016) describes the dorsal ACC as a region of response selection, integrating action values with action outcomes, whereas the anterior insula employs this information for adjusting behavioral responses. The MFG, in turn, counteracts aggressive impulses (Achterberg et al., 2016), and enhanced connectivity of amygdala with MFG and dACC has been associated with reduced anger states after virtual aggression (Klasen et al., 2013). However, the present study did not find any significant impact of ATD on these neural systems. Instead, the findings indicate a reduced synchronization between affective and empathy networks as an effect of the serotonergic challenge. Thus, the role of serotonin in aggression may be less a modulation of the emotional response, but rather an attenuation of empathy with the victim.

Moreover, our data delivered evidence that connectivity in serotonergic aggression networks varies with personality traits. Specifically, ATD effects on amygdala connectivity with IFG and OFC varied as a function of trait aggression. ATD modulation of OFC and IFG has been shown previously for response inhibition, confirming the role of prefrontal serotonergic activity for inhibitory control (Rubia et al., 2005). Accordingly, the PFC-amygdala system plays an essential role in emotion regulation (Eden et al., 2015). Impairments of this regulation system are associated with trait aggression (Raine and Yang, 2006). In a similar vein, reduced gray matter in amygdala and PFC regions including the OFC have consistently been associated with trait aggression as well (Ermer et al., 2012). Functional neuroimaging studies corroborate the relevance of this frontolimbic system for the control of aggressive impulses; specifically, reduced IFG-amygdala connectivity is frequently observed in overly aggressive individuals (see Bogerts et al., 2018, for a review). Similar findings have also been obtained for the OFC; amygdala-OFC connectivity shows a negative association with trait anger (Fulwiler et al., 2012). Although encompassing the same anatomical regions, our findings revealed an opposite pattern: trait aggression was positively associated with PFC-amygdala connectivity in the placebo condition. These findings may be explained by the neurobiology of video game violence. Aggression-enhancing effects of violent games are more pronounced in individuals with high trait aggression (Peng et al., 2008). However, aggressive actions in the game are characterized by enhanced coupling in regulatory PFC-amygdala circuits, indicating an emotion regulation in favor of the cognitive game task (Mathiak and Weber, 2006; Klasen et al., 2013). Thus, it is likely that individuals with higher trait aggression required a stronger frontolimbic emotion control for successfully performing violent game actions.

Finally, there are first indications for genotype influences on serotonergic aggression networks. In our study, carriers of the low expressing risk allele exhibited higher susceptibility of the PFC-amygdala system to ATD than high expressing allele carriers. Higher vulnerability of fronto-amygdalar regulation systems in *MAOA*-L carriers have previously been discussed as a risk factor for aggression. An established model suggests aggression-related genotype differences in amygdala control via anterior cingulate and ventromedial PFC (Buckholtz et al., 2008); however, this model has recently been challenged (Klasen et al., 2018). Our data, in turn, suggest a genotype-specific involvement of left MFG, which is in line with earlier findings on *MAOA* effects in emotion processing (Alia-Klein et al., 2009). Only in *MAOA*-L carriers, ATD impact on MFG-amygdala connectivity varied as a function of trait aggression. Thus, brain aggression networks seem to depend on *MAOA* genotypes, which is in line with recent findings (Klasen et al., 2018). In analogy to the model by Buckholtz et al. (2008), we suggest the left MFG as a *MAOA*-L-specific supplementary node for serotonergic amygdala regulation. Disruption of this regulatory system may accordingly be associated with increased risk of aggression in *MAOA*-L carriers. However, albeit a clear trend was observed, genotype differences in this analysis are descriptive.

A final interesting perspective comes from a recent investigation by Manca et al. (2018). This study revealed a role of a second *MAOA* gene polymorphism (dVNTR) on MAO-A expression. Specifically, the two VNTRs differentially affect the expression of different MAO-A isoforms. As a whole, there seem to be mechanistic interactions between the two VNTRs (Manca et al., 2018). The associations of dVNTR and dVNTR-uVNTR interactions with behavioral variables, however, have still to be established. Future studies should take these novel findings into account, which will have an impact on sample sizes as well. Given the observed frequencies of observed dVNTR-uVNTR repeat combinations (cf. Manca et al., 2018), larger samples will be required to investigate interactions between the two polymorphisms.

## Conclusion

The present study highlights the role of serotonin in brain aggression networks. Specifically, as revealed by ATD challenge, serotonergic projections connecting limbic areas (amygdala) to empathy networks may influence the emotional assessment of aggressive actions. Additionally, amygdala connectivity with MFG and OFC is negatively correlated with trait aggression, which may constitute a supplementary emotion regulation system. Enhanced vulnerability of this system may foster aggression in *MAOA* risk allele carriers.

## Ethics Statement

This study was carried out in accordance with the recommendations of the Code of Ethics of the Word Medical Association with written informed consent from all subjects. All subjects gave written informed consent in accordance with the Declaration of Helsinki. The protocol was approved by the Ethics Committee at the RWTH Aachen Faculty of Medicine.

## Author Contributions

KM, FZ, and RW designed the paradigm. MK, DW, and PE collected the data. MK analyzed the neuroimaging data. TE and KZ conducted the genotyping. KM and RW supported data analysis and interpretation. MK wrote and all co-authors corrected the manuscript. All authors gave final approval of the version to be published.

## Conflict of Interest Statement

The authors declare that the research was conducted in the absence of any commercial or financial relationships that could be construed as a potential conflict of interest.
